# TTP‐Like Syndrome and Subsequent Non‐Aneurysmal Subarachnoid Hemorrhage in HbSC Disease: A Case Report

**DOI:** 10.1002/ccr3.73501

**Published:** 2026-09-10

**Authors:** Benjamin Vieten, Jan Vorwerk, Jan Morf, Alice Dauth, Stefan Sondermann, Peter Schramm, Tobias Graf, Nikolas von Bubnoff, Lorenz Oelschläger, Cyrus Khandanpour

**Affiliations:** ^1^ Department of Hematology and Oncology, University Cancer Center Schleswig‐Holstein (UCCSH), University Hospital Schleswig‐Holstein (UKSH) University of Luebeck Luebeck Germany; ^2^ Department of Medicine I, University Hospital Schleswig‐Holstein (UKSH) University of Luebeck Luebeck Germany; ^3^ Department of Neurosurgery, University Hospital Schleswig‐Holstein (UKSH) University of Luebeck Luebeck Germany; ^4^ Department of Neuroradiology, University Hospital Schleswig‐Holstein (UKSH) University of Luebeck Luebeck Germany; ^5^ Department of Cardiology, Angiology and Intensive Care Medicine, University Hospital Schleswig‐Holstein (UKSH) University of Luebeck Luebeck Germany; ^6^ German Center for Cardiovascular Research (DZHK) Partner Site North Luebeck Germany; ^7^ Department of Hematology and Oncology Klinikum Oldenburg Oldenburg Germany; ^8^ University Medicine Oldenburg Carl von Ossietzky University Oldenburg Germany

**Keywords:** fat embolism syndrome, HbSC, sickle cell disease, subarachnoid hemorrhage, TTP, TTP‐like syndrome

## Abstract

Sickle cell disease (SCD) with hemoglobin‐ (Hb‐) SC genotype is often considered a milder SCD variant, yet life‐threatening complications can occur. A 26‐year‐old man with HbSC disease presented with an infection triggered vaso‐occlusive crisis (VOC), acute chest syndrome (ACS), severe thrombocytopenia, Coombs‐negative hemolysis with schistocytes, acute kidney injury, and markedly elevated lactate dehydrogenase (LDH) in the setting of *Klebsiella pneumoniae* sepsis. The resulting severe thrombotic microangiopathy‐ (TMA‐) like phenotype initially raised concern for thrombotic thrombocytopenic purpura (TTP). However, ADAMTS13 activity was not significantly reduced, arguing against classical TTP. The episode was interpreted as a TTP‐like syndrome, whereas fat embolism syndrome (FES) remained an important differential diagnosis. The patient received red‐cell exchange, plasma exchange, caplacizumab, antibiotics, and dialysis, followed by hematologic and renal recovery. Months later, he developed a non‐aneurysmal infratentorial subarachnoid hemorrhage (SAH) with obstructive hydrocephalus requiring external ventricular drainage (EVD) and posterior fossa decompression, resulting in severe persistent neurological disability. The case, therefore, highlights the severity of HbSC disease and the challenge of urgent treatment decisions under substantial diagnostic uncertainty when a TMA‐like presentation overlaps with other severe SCD complications such as FES.

## Key Clinical Message

Although HbSC disease is often considered a milder SCD variant, life‐threatening TMA‐like presentations and non‐aneurysmal subarachnoid hemorrhage can occur. This case highlights the need for rapid treatment decisions under diagnostic uncertainty when a presumptive TTP‐like syndrome overlaps clinically with fat embolism syndrome.

## 1 Introduction

Sickle cell disease (SCD) is one of the most common inherited hemoglobinopathies worldwide. It is caused by a point mutation in the β‐globin gene (Glu6Val), which reduces the deformability of erythrocytes under deoxygenated conditions. Over time, SCD can lead to life‐shortening complications. SCD comprises a spectrum of genotypes. Although hemoglobin‐ (Hb‐) SS is considered the most severe, compound heterozygous forms such as HbSC are generally regarded as milder. Globally, approximately 7.7 million people and 500,000 newborns annually are affected by SCD, most of whom live in Sub‐Saharan Africa [1]. Among SCD genotypes, HbSC is very frequent. Population cohorts, such as the United States SCDIC registry, show that approximately 22% of patients aged 15–45 with SCD have HbSC [2]. Although HbSC has traditionally been considered milder than HbSS, increasing evidence demonstrates that it is associated with substantial morbidity including acute chest syndrome (ACS), splenomegaly, and avascular necrosis [2]. In rare cases, devastating complications such as thrombotic microangiopathy (TMA) or intracranial hemorrhage have been reported [3, 4, 5, 6].

The term thrombotic thrombocytopenic purpura‐ (TTP‐) like syndrome has been used to describe TMA‐like presentations that clinically resemble TTP but occur without severe ADAMTS13 deficiency. In suspected TTP, urgent therapeutic plasma exchange is a key component of treatment [7]. Similar rescue strategies using plasma and red‐cell exchange have been reported in patients with HbSC‐associated TTP‐like presentations [8]. Randomized trials have shown that treatment with caplacizumab, a nanobody targeting the A1 domain of von Willebrand factor (vWF), in acquired TTP can accelerate platelet recovery and reduce exacerbations [9]. Fat embolism syndrome (FES) represents an important differential diagnosis in SCD, particularly in HbSC disease, as it can lead to respiratory failure, thrombocytopenia, and neurological manifestations, all similar to a TTP‐like presentation [10].

Hemorrhagic cerebrovascular events, such as subarachnoid hemorrhage (SAH), are rare complications of SCD and tend to occur at younger ages [4]. A study conducted in the USA found that among 468,070 SCD admissions, only 0.18% were complicated by intracranial bleeding (ICB) [5]. The inpatient mortality rate for SCD with ICB was 25.4%, compared to 0.6% for SCD without ICB [5]. In such cases, in addition to immediate neurosurgical care, perioperative management with a focus on transfusion strategies is important. Exchange transfusion to reduce HbS levels is recommended to optimize cerebral oxygen delivery and reduce complications [2]. Despite neurosurgery and other treatments, brainstem injury due to SAH cannot always be prevented and may result in poor outcomes such as tetraparesis and dysarthria.

In this case report, we describe the diagnostic and therapeutic challenges of a presumed TTP‐like syndrome and subsequent infratentorial SAH in a patient with HbSC disease.

## 2 Case History and Methods

A 26‐year‐old man with a confirmed HbSC genotype by Hb electrophoresis was known for a history of SCD, recurrent vaso‐occlusive crises (VOC), and avascular necrosis of the right femoral head. He had taken hydroxyurea intermittently and had not received long‐term transfusion therapy. His mother was diagnosed with SCD too.

**FIGURE 1:**
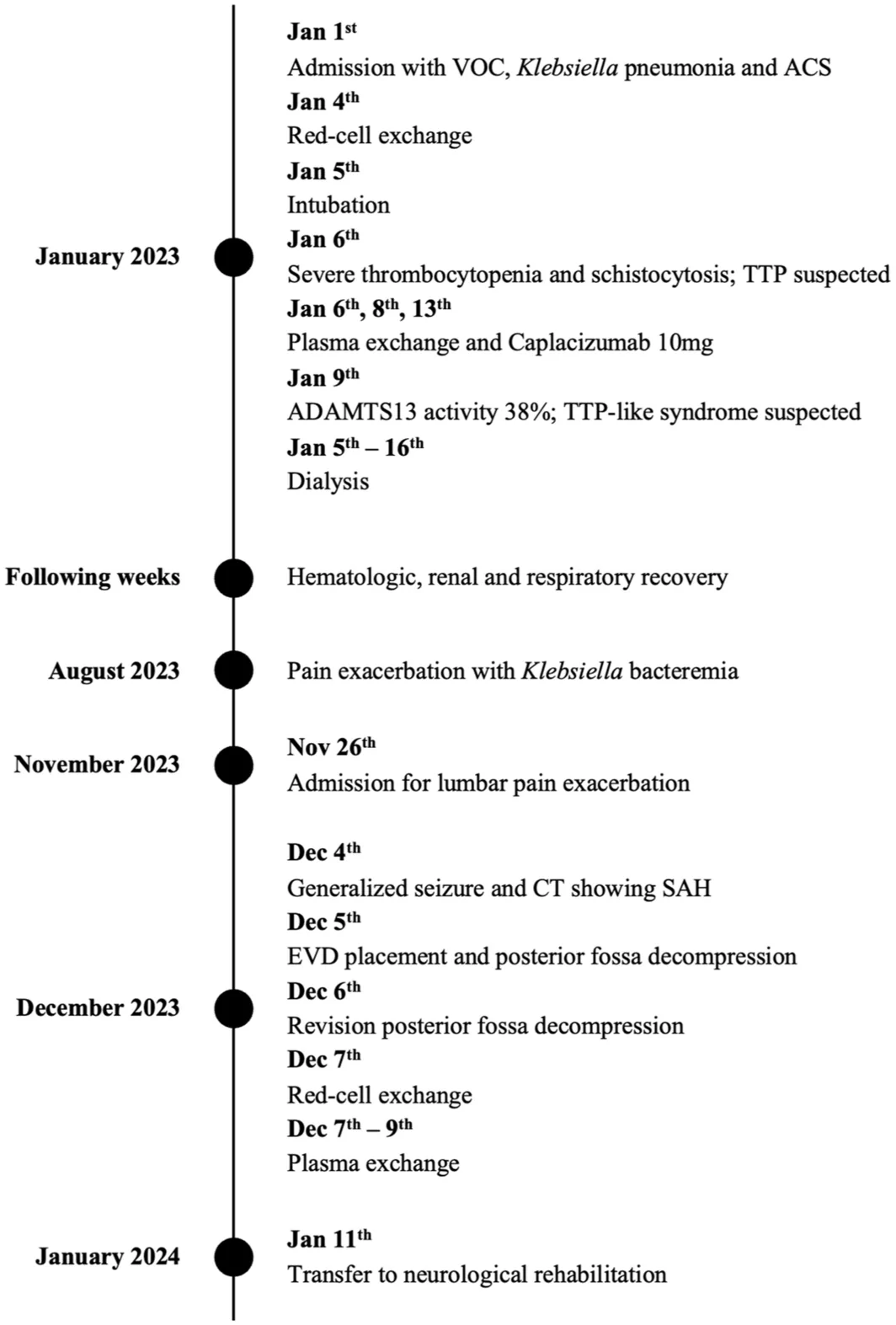
Timeline of major clinical events.

The subsequent clinical course is summarized in Figure 1. In January 2023, the patient presented to the emergency department with chest pain, high fever, tachycardia, dyspnea, and chills for 2 days. Elevated inflammatory markers including procalcitonin (PCT) and C‐reactive protein (CRP), and chest computed tomography (CT) revealing bilateral patchy consolidations suggested ACS as a severe complication of HbSC together with pneumonia and sepsis, which later was supported by the growth of *Klebsiella pneumoniae* in blood cultures. Empiric piperacillin–tazobactam and an augmented analgetic regimen were initiated, and the patient was admitted to the intensive care unit (ICU) on Day 1. Despite the taken measures, the condition deteriorated into respiratory decompensation over the following days. Sufficient oxygenation was maintained through high‐flow oxygen therapy and non‐invasive ventilation. On Day 4, after initially declining the procedure, the patient underwent urgent red‐cell exchange transfusion to reduce the sickle Hb fraction and improve oxygen delivery in the setting of ACS. Progressive respiratory failure subsequently required invasive mechanical ventilation from Day 5.

CT angiography of the chest and abdomen and magnetic resonance imaging (MRI) including magnetic resonance angiography (MRA) of the supraaortic region performed during the first 4 days after admission demonstrated splenomegaly, avascular necrosis of the right femoral head, and pulmonary infiltrates consistent with pneumonia. Infarcts of the spleen could not be ruled out, whereas no signs of embolic events in the lung, intestine, or brain were found. Venous thrombosis without occlusion in both iliac veins was detected.

**FIGURE 2:**
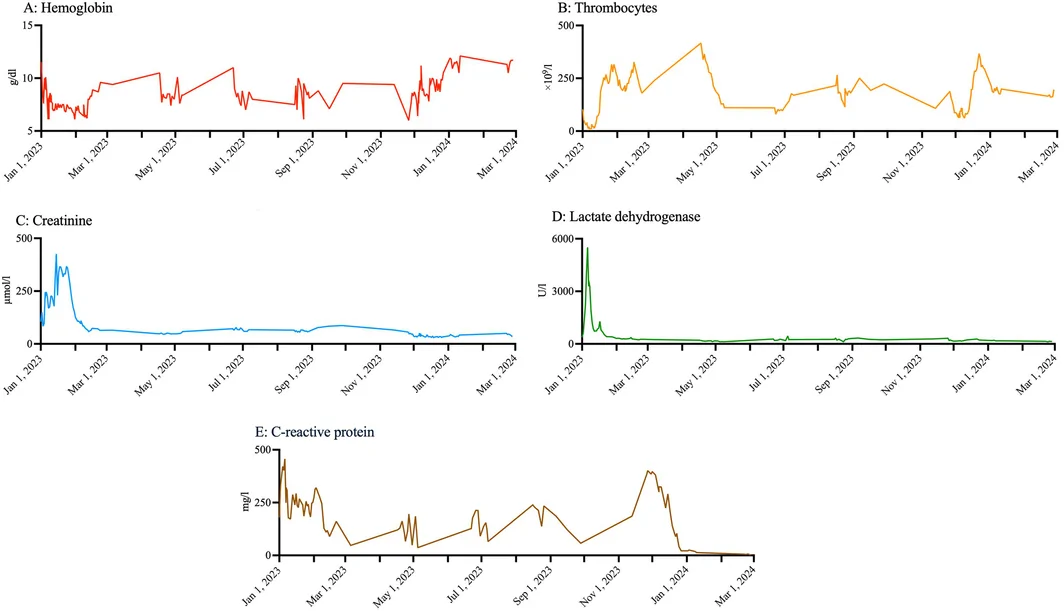
Laboratory values during the course of the disease. (A) Hemoglobin (Hb); (B) thrombocytes; (C) creatinine; (D) lactate dehydrogenase (LDH); and (E) C‐reactive protein (CRP). Throughout medical history, decisions were made based on the development of laboratory values and clinical signs. Reference values for male adult [11]: Hb 13.5–17.5 g/dL; Thrombocytes 150–350 × 10^9^/L; Creatinine < 133 μmol L; LDH 100–190 U/L; CRP 0.08–3.10 mg/L.

On Day 6, thrombocytopenia of unknown origin became a concern when laboratory results showed thrombocytes at 13 × 10^9^/L, lactate dehydrogenase (LDH) of 5517 U/L, decreased fibrinogen, and elevated D‐dimer (Figure 2B,D). Particularly notable were schistocytes, which increased during clinical deterioration from initially 2‰ to 5‰ on Day 6 and reached a maximum value of 9‰ on Day 9. Coombs‐negative hemolysis, acute kidney injury requiring dialysis, and an altered mental status before intubation together with the thrombocytopenia and schistocytosis raised concern for a TMA. The severe TMA‐like phenotype and a high calculated PLASMIC score raised concern for TTP while ADAMTS13 testing was still pending. Because severe ADAMTS13 deficiency could not yet be excluded, TTP‐directed treatment was initiated before the ADAMTS13 result became available.

Therapeutic plasma exchange with a volume of 3000 mL was performed on Days 6, 8, and 13. In addition, caplacizumab was initiated. A 10 mg intravenous dose was given before the first plasma exchange, whereas two further doses were administered subcutaneously after plasma exchanges. Furthermore, the patient required intermittent dialysis therapy from Day 5 to Day 16 for renal failure.

The first ADAMTS13 result became available on Day 9 and showed an activity of 38% (reference: 40%–130%). The absence of severe ADAMTS13 deficiency argued against classical TTP. In view of the persistent TMA‐like clinical phenotype and after considering other differential diagnoses as FES and other secondary TMAs during multidisciplinary review, the episode was interpreted as a presumptive TTP‐like syndrome. Given the absence of severe ADAMTS13 deficiency and the subsequent clinical improvement, caplacizumab was not continued for the usual 30‐day post‐plasma‐exchange treatment period used in immune‐mediated TTP.

A positron emission tomography (PET‐) CT scan performed to identify a potential infectious focus revealed patchy hypermetabolic areas within the bone marrow (BM). Histopathologic examination of a BM biopsy demonstrated reactive BM without overt necrosis or other pathologies.

Over the next few weeks, laboratory and clinical parameters improved. Recovery of the thrombocyte count, a decrease in LDH, CRP, and creatinine with return of urine output indicated hematologic and renal recovery (Figure 2A–E). The recovery of thrombocytes enabled initiation of anticoagulation therapy for the iliac and inferior vena cava thromboses. At the same time, pathogen‐negative blood cultures and declining CRP and PCT levels allowed stepwise narrowing of antibiotics. The patient was discharged after several weeks on anticoagulation, analgesics, and hydroxyurea.

During spring and summer of 2023, the patient had several readmissions for SCD‐related complications including VOC. In August he was readmitted for *Klebsiella pneumoniae* bacteremia.

**FIGURE 3:**
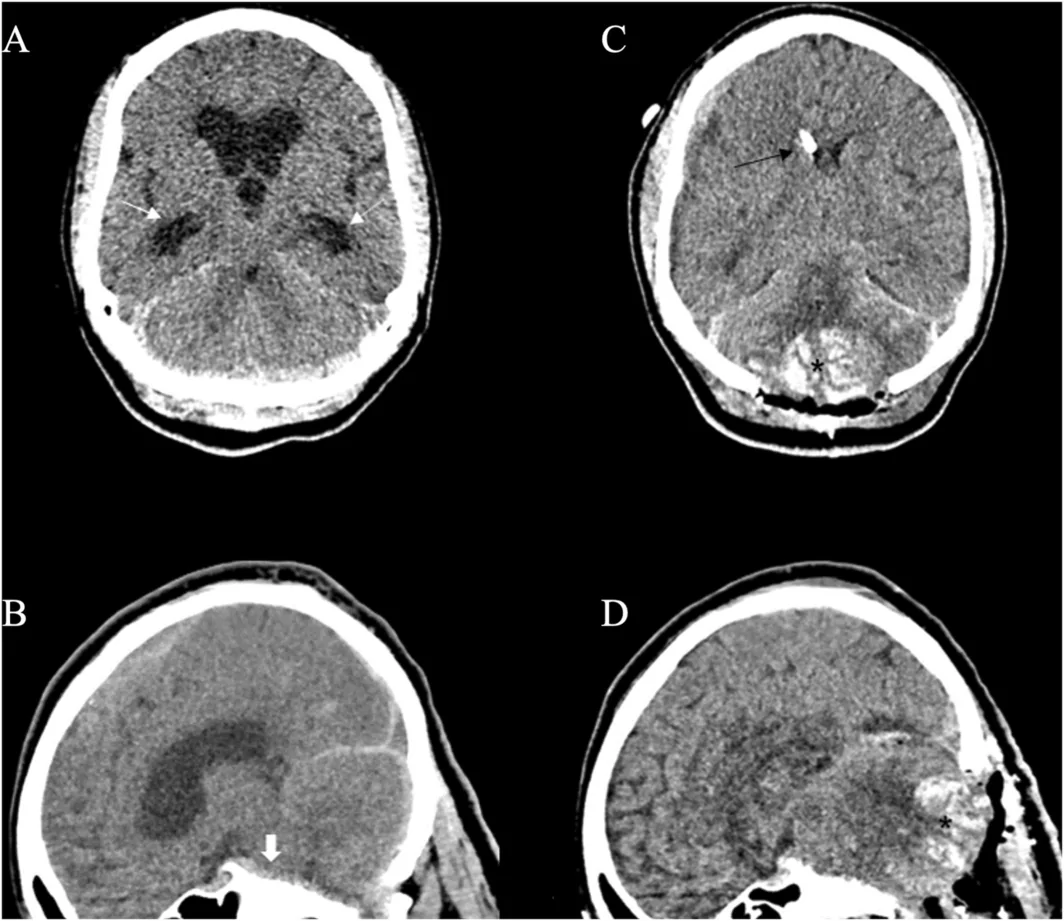
Cranial computed tomography (cCT). (A, B) Initial imaging with obstructive hydrocephalus due to infratentorial subarachnoid hemorrhage (SAH) (A white arrows: Temporal horn enlargement; B bold white arrow: SAH in the prepontine cisterna). (C, D) Imaging after posterior fossa decompression and external ventricular drainage (EVD) (C black arrow: Ventricular catheter tip; C, D asterisk: Cerebellar decompression hemorrhages).

At the end of November 2023, the patient presented with acute lumbar pain exacerbation during a sickle cell crisis. On December 4th, the patient experienced a generalized seizure. Non‐contrast CT revealed infratentorial SAH with developing obstructive hydrocephalus (Figure 3A,B). CT angiography and digital subtraction angiography (DSA) of the cerebral vessels did not identify an intracranial aneurysm or another potential bleeding source. During angiography, the respiratory condition deteriorated requiring intubation and transfer to the ICU. The patient subsequently developed anisocoria. CT imaging revealed increasing hydrocephalus, cerebellar swelling, and incipient tonsillar herniation. Neurosurgery proceeded with emergent external ventricular drainage (EVD) to relieve intracranial pressure, followed by posterior fossa decompression. Postoperative CT imaging showed extensive cerebellar hemorrhage and progressive cerebellar swelling, requiring revision posterior fossa decompression on December 6th (Figure 3C,D). Later, persistent hydrocephalus required replacement of the EVD and subsequent ventriculoperitoneal shunt placement.

On December 7th, red‐cell exchange transfusion was performed to reduce the sickle Hb fraction. Because of renewed clinical and laboratory deterioration resembling the initial episode, therapeutic plasma exchange was subsequently performed on December 7th, 8th, and 9th. Histopathology showed clotted blood with fibrin and numerous sickled erythrocytes, without vascular malformation, neoplasia, or amyloid angiopathy. The SAH, obstructive hydrocephalus, tonsillar herniation, and subsequent bleeding complications resulted in severe neurological injury despite immediate neurosurgical intervention. The patient showed a delayed waking response, dysphagia, persistent tachycardia, and a prolonged period of respiratory support, prompting tracheostomy.

In early January 2024, HbS polymerization inhibitor voxelotor was initiated as hemolytic anemia persisted despite hydroxyurea titration to the maximum dose. Subsequently, Hb increased modestly and transfusion requirements decreased (Figure 2A).

After these events, routine laboratory testing showed a more stable pattern than during the initial critical illness, with preserved renal function and no recurrence of fulminant multiorgan failure. He was transferred to neurorehabilitation, where he recovered partially.

## 3 Outcome

By March 2024, at his last hospital contact, he presented awake, with arm‐predominant tetraparesis, dysarthria, bilateral oculomotor paresis, tracheostomized, and entirely care‐dependent. He now resides in a long‐term care facility. He has regained limited speech but remains affected by severe cognitive and motor impairment, including retrograde amnesia.

## 4 Discussion

Classical TTP is a well‐defined TMA caused by either congenital or acquired severe ADAMTS13 deficiency. The term TTP‐like syndrome has been used for clinical presentations resembling TTP in critically ill patients without severe ADAMTS13 deficiency [12]. One proposed hypothesis for the pathogenesis of TTP‐like syndrome is the downregulation of CD59 in endothelial cells and consequently the activation of the complement system in critically ill patients. This in turn leads to platelet activation and excessive endothelial exocytosis of ultra‐large vWF multimers. The resulting imbalance between increased vWF and ADAMTS13 activity can lead to the formation of platelet‐rich microthrombi resembling those observed in TTP [12].

In the present case, severe *Klebsiella pneumoniae* infection and sepsis may have precipitated marked endothelial activation and a secondary TMA‐like phenotype in the setting of VOC and ACS. Sickle cell crisis is characterized by marked endothelial activation and increased release of vWF multimers [13]. Accordingly, the markedly elevated vWF antigen level of 686% (reference: 50%–160%) was consistent with this inflammatory endothelial response [14]. This plausible pathophysiological mechanism, together with severe thrombocytopenia, Coombs‐negative hemolysis with schistocytes, acute kidney injury, and elevated LDH, supported the presence of a severe TMA‐like phenotype. The severity of this constellation exceeded that expected from an uncomplicated VOC [2, 15]. ADAMTS13 activity was moderately reduced at 38%, arguing against classical TTP. In the context of the fulminant clinical presentation, we favored the presumptive diagnosis of a TTP‐like syndrome. A similar TTP‐like presentation in HbSC disease without severe ADAMTS13 deficiency has been described by Kodali et al. [8].

Other causes of thrombocytopenia were considered as well. Heparin‐induced thrombocytopenia (HIT) became unlikely after negative antibody testing. Immune thrombocytopenia (ITP) was considered less likely as the thrombocytopenia was accompanied by schistocytosis, Coombs‐negative hemolysis, and acute kidney injury, favoring a microangiopathic process. Sepsis‐associated coagulopathy represented an additional competing diagnosis given the severe *Klebsiella* sepsis, decreased fibrinogen, and elevated D‐dimer. However, the progressive schistocytosis, Coombs‐negative hemolysis, and multiorgan involvement raised concern for a TMA‐like process.

An important differential diagnosis considered was FES secondary to BM infarction. FES is particularly relevant in patients with HbSC disease and can present with respiratory failure, thrombocytopenia, anemia, renal dysfunction, and neurological impairment, thereby mimicking TTP [10]. These features were present in the patient described, making FES an important competing diagnosis. CT pulmonary and abdominal angiography showed no evidence for pulmonary embolism. However, this did not exclude microvascular fat embolization. The absence of overt BM necrosis on biopsy did not provide histopathological support for FES. These findings could not reliably exclude microvascular fat embolization or BM infarction. Therefore, FES could not be definitively excluded.

The initial suspicion of TTP guided urgent treatment decisions while ADAMTS13 results were pending. In TTP, therapeutic plasma exchange is the cornerstone of treatment according to current International Society on Thrombosis and Hemostasis (ISTH) guidelines [7]. Furthermore, reports have described successful treatment of a similar HbSC‐associated TTP‐like syndrome with plasma and red‐cell exchange, as well as the use of caplacizumab in acquired TTP [3, 8]. The addition of caplacizumab was consistent with a double‐blind, controlled trial demonstrating faster platelet recovery and a 67% reduction in TTP recurrence compared with placebo in acquired TTP [9]. Importantly, plasma and red‐cell exchange is also a principal treatment for SCD‐associated FES. The rapid normalization of platelet counts and decline in LDH suggested effective therapy. Because red cell exchange, plasma exchange, caplacizumab, antimicrobial treatment, dialysis, and supportive care were administered in close temporal proximity, the improvement could not be attributed to a single intervention.

During the subsequent months, recurrent bacteremia and persistent inflammatory activation were apparent, reflected by repeated CRP and thrombocyte surges. Despite re‐titrated hydroxyurea, LDH and bilirubin remained above baseline, consistent with ongoing hemolysis, which may have contributed to continued endothelial stress. Chronic hemolysis is associated with nitric oxide depletion, which in turn promotes oxidative stress and alters blood flow, hemostasis, and angiogenesis [16]. Repeated inflammatory activation can promote intimal hyperplasia and vascular remodeling [17]. Collectively, these processes provide a biologically plausible background of weakened vascular vulnerability in this patient.

The later development of infratentorial SAH can be interpreted in the context of the possible vascular vulnerability. Hemorrhagic stroke is a rare complication of SCD [4, 5, 6]. In our case, neither imaging nor histopathology identified an aneurysm or malformation. Therefore, sickle‐associated vascular vulnerability remains one possible explanation for the hemorrhage. Cerebrovascular disease in SCD is multifactorial. Chronic endothelial activation, hemolysis‐driven nitric oxide depletion, and inflammatory vascular remodeling may have contributed to the hemorrhagic SAH in this patient [16, 17]. The coexistence of a prior TTP‐like crisis and subsequent SAH in this case may therefore have occurred on a shared background of systemic endothelial dysfunction and sickle‐associated vascular vulnerability. However, a direct pathophysiological link between the two events cannot be established.

The infratentorial location was decisive for the outcome. Posterior fossa hemorrhage rapidly led to obstructive hydrocephalus and brainstem compression, necessitating emergent EVD and decompression. Despite prompt neurosurgical intervention and exchange transfusion to minimize perioperative sickling risk, severe neurological injury nevertheless occurred, including arm‐predominant tetraparesis, dysarthria, dysphagia, and oculomotor paresis. In other reports of SCD‐associated stroke, similarly devastating outcomes have been described [18].

From a longitudinal perspective, this case demonstrates the abrupt transition from chronic HbSC disease to irreversible neurological disability within 1 year. Prior to 2023, the patient lived independently despite recurrent VOC. Despite recovery from the initial TMA‐like episode, he subsequently developed devastating cerebrovascular disease. Ongoing endothelial stress and inflammatory activity may have contributed to vascular vulnerability.

## 5 Conclusion

Overall, this case underscores the potential for HbSC disease to manifest with severe TMA‐like and hemorrhagic vascular events within the same disease course. The laboratory evolution and clinical trajectory suggest systemic endothelial dysfunction as a possible unifying pathophysiological background. The need for rapid treatment decisions under diagnostic uncertainty represents a major clinical challenge. Even with rapid interdisciplinary and multimodal management, life‐threatening neurological complications can occur, highlighting the potential severity of HbSC disease.

## Author Contributions

**Benjamin Vieten:** writing – original draft, conceptualization, investigation. **Jan Vorwerk:** writing – original draft, conceptualization, investigation. **Jan Morf:** writing – review and editing, investigation. **Alice Dauth:** visualization, writing – original draft. **Stefan Sondermann:** visualization. **Peter Schramm:** visualization, writing – review and editing. **Tobias Graf:** writing – review and editing, investigation. **Nikolas von Bubnoff:** writing – review and editing, investigation. **Lorenz Oelschläger:** writing – review and editing, project administration, supervision. **Cyrus Khandanpour:** writing – review and editing, project administration, supervision.

## Funding

The authors have nothing to report.

## Consent

Written informed consent for publication of this case report and any accompanying images was obtained from the patient's wife.

## Conflicts of Interest

The authors declare no conflicts of interest.

## Data Availability

The data that support the findings of this study are available from the corresponding author upon reasonable request.
